# Towards a Meaningful 3D Map Using a 3D Lidar and a Camera

**DOI:** 10.3390/s18082571

**Published:** 2018-08-06

**Authors:** Jongmin Jeong, Tae Sung Yoon, Jin Bae Park

**Affiliations:** 1 School of Electrical and Electronic Engineering, Yonsei University, Seoul 03722, Korea; jeong6560@yonsei.ac.kr; 2 Department of Electrical Engineering, Changwon National University, Changwon-Si 51140, Korea; tsyoon@changwon.ac.kr

**Keywords:** 3D Lidar, large-scale mapping, map refinement, moving vehicle removal, semantic mapping, semantic reconstruction

## Abstract

Semantic 3D maps are required for various applications including robot navigation and surveying, and their importance has significantly increased. Generally, existing studies on semantic mapping were camera-based approaches that could not be operated in large-scale environments owing to their computational burden. Recently, a method of combining a 3D Lidar with a camera was introduced to address this problem, and a 3D Lidar and a camera were also utilized for semantic 3D mapping. In this study, our algorithm consists of semantic mapping and map refinement. In the semantic mapping, a GPS and an IMU are integrated to estimate the odometry of the system, and subsequently, the point clouds measured from a 3D Lidar are registered by using this information. Furthermore, we use the latest CNN-based semantic segmentation to obtain semantic information on the surrounding environment. To integrate the point cloud with semantic information, we developed incremental semantic labeling including coordinate alignment, error minimization, and semantic information fusion. Additionally, to improve the quality of the generated semantic map, the map refinement is processed in a batch. It enhances the spatial distribution of labels and removes traces produced by moving vehicles effectively. We conduct experiments on challenging sequences to demonstrate that our algorithm outperforms state-of-the-art methods in terms of accuracy and intersection over union.

## 1. Introduction

**Figure 1:**
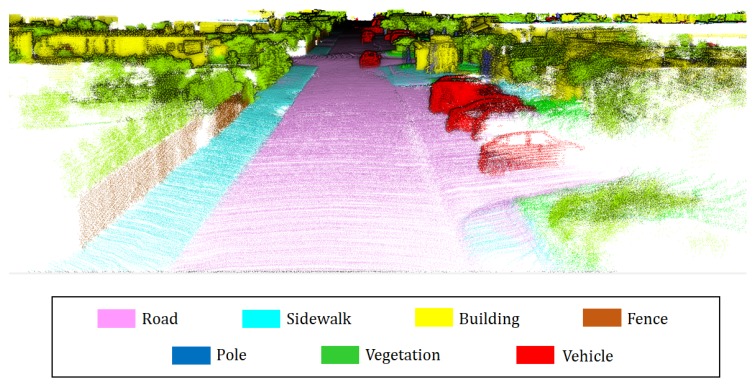
Example of a semantic 3D map generated by the proposed method.

As demands for Lidar system increase, a lot of research including airborne lidar studies [1,2,3] and mobile lidar studies [4,5] have been conducted. Among the various research topics, research on semantic 3D mapping is an important field in various robotics applications such as autonomous robots [6] and robot interaction [7]. Semantic 3D mapping implies reconstructing the real environment into 3D space and also embedding semantic information in a map. A semantic 3D map simultaneously stores location information and semantics of the environment as shown in Figure 1. Existing 3D maps that contain only geometry information involve limitations in terms of enabling robots to perform high-level tasks or to better understand a surrounding scene, and thus, a 3D map that includes semantic information is required. Previously, several studies focused on simultaneously localization and mapping (SLAM) [8,9,10] to reconstruct geometry information, and they were applied to various robotics applications such as robot navigation [11], and robot control [12]. Recently, with the development of semantic segmentation technology, interest in semantic 3D mapping is increasing and several related studies were introduced [13,14,15]. However, the combination of a semantic segmentation with a 3D mapping is still a challenging topic.

First of all, a fundamental part for semantic mapping is semantic segmentation. Studies on semantic segmentation can be divided into 3D semantic segmentation and 2D semantic segmentation according to the spatial domain in which the algorithm is performed. 3D semantic segmentation takes a point cloud directly as an input and assigns a semantic label to each point. Recently, several researchers modified 2D convolutional neural network (CNN) structure and applied it to 3D semantic segmentation such as PointNet [16] and SEGCloud [17]. However, they require a long computation time as they are performed in 3D space. Furthermore, there is the limitation that the performance of 3D semantic segmentation is inferior to that of 2D semantic segmentation. Two-dimensional semantic segmentation involves assigning a semantic label to each pixel in an image, and a lot of studies have been conducted since it is considered as an important field in computer vision. Several researchers addressed semantic labeling by using a probabilistic model such as Markov Random Field (MRF) and Conditional Random Field (CRF) [18,19]. Recently, with respect to the strength of development of CNN, several studies solved the problem of semantic segmentation by using CNN, and they exhibited significant performance improvements. A representative approach was the FCN of Shelhamer et al. [20]. They introduced an upsampling layer termed as transposed convolutional layer. Subsequently, several outstanding network architectures for 2D semantic segmentation were developed such as ENet [21], SegNet [22], DilatedNet [23], and RefineNet [24]. Specifically, RefineNet motivated by ResNet exhibited fine and coarse segmentation results since it receives information from the different resolutions via various-range connections [24]. In this study, we chose the RefineNet as a 2D semantic segmentation tool because it exhibits the best performance among state-of-the-art methods and is open source.

Recently, several studies on semantic 3D mapping were introduced given the evolution of 2D semantic segmentation and the creation of a few open source SLAM algorithms. Chen et al. achieved semantic mapping in an indoor environment by integrating ORB-SLAM and CRF-RNN [25]. In [26], a 3D labeling method was proposed by combining dense pairwise 3D CRF and the Bayesian update scheme, and thereby a 3D semantic map for an indoor scene was successfully generated. Furthermore, in [14], semantic 3D mapping was performed by using deep learning-based segmentation and LSD-SLAM system and subsequently correspondences between keyframes were used to transfer 2D semantic information into 3D grids. Sengupta et al. utilized octree representation to efficiently perform semantic mapping and embedded hierarchical robust $P^{N}$ MRF for semantic segmentation [27]. In [15], a dense 3D map was generated by combining ORB-SLAM and ELAS and optimizing 3D grid labels by using CNN-based segmentation and the CRF model with a higher order, and thereby creating a dense semantic 3D map. Additionally, in [13], an incremental 3D map was constructed from a stereo camera, and semantic labeling was performed by using a random forest with CRF. However, existing approaches focused on the camera-based method and suffered from a variety of limitations. For example, they are unable to adapt to large-scale scenarios due to high computational complexity and to create accurate 3D maps due to depth inaccuracy. Additionally, it is difficult to generate a 3D map in an environment with insufficient features. Furthermore, existing studies did not consider removing traces of moving vehicles remaining in a map. These traces are useless information, and thus, it is necessary to remove them.

In this study, we developed semantic 3D mapping by fusing a 3D Lidar with a camera. Our goal is to create a semantic 3D map with the following seven labels: *road*, *sidewalk*, *building*, *fence*, *pole*, *vegetation*, and *vehicle*. We consider seven labels because the objects corresponding to these labels occupy most urban environments. With respect to the 3D reconstruction in our method, a GPS and an IMU are utilized to estimate the odometry of our system, and the point cloud obtained from a 3D Lidar is transformed by using estimated odometry. With respect to semantic segmentation, the CNN-based 2D semantic segmentation is used to obtain spatial distribution for seven labels. Three-dimensional semantic segmentation is not suitable for large-scale environments because it is not only inferior in performance but also takes a long time to implement compared to 2D semantic segmentation. Therefore, we exploit 2D semantic segmentation instead of 3D semantic segmentation. In order to integrate semantic information with the point cloud, we developed incremental semantic labeling that transfers pixel label distributions on an image to 3D grid space. However, traces of moving vehicles remain on a generated semantic map, and the map is not accurate due to errors from 2D semantic segmentation and semantic labeling. In order to solve these problems, we developed a process of map refinement that is performed in a batch. We use the map refinement to remove traces and effectively rectify the spatial distribution of labels.

In summary, our main contributions are as follows:

- We developed a semantic 3D mapping algorithm suitable for large-scale environments by combining a 3D Lidar with a camera.
- We presented incremental semantic labeling including coordinate alignment, error minimization, and semantic information fusion to enhance the quality of a semantic map.
- We developed map refinement to remove traces and improve the accuracy of a semantic 3D map.
- We improved 3D segmentation accuracy over state-of-the-art algorithms on the KITTI dataset.

The remainder of the study is organized as follows: In Section 2, the proposed algorithm is described in detail. Section 3 details the experiments against other state-of-the-art methods and discusses the experimental results. The conclusions are presented in Section 4.

## 2. Approach

**Figure 2:**
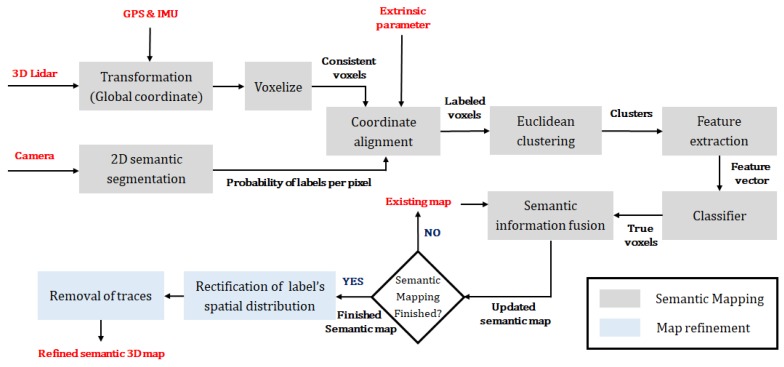
Flowchart of the semantic 3D mapping method.

Our system combines a 3D Lidar and a camera to create a semantic 3D map that is designed for urban environments. Our semantic map includes seven labels (*road*, *sidewalk*, *car*, *building*, *vegetation*, *pole*, *fence*), and this is reasonable because objects corresponding to these labels occupy most of the environment. As shown in Figure 2, the proposed method consists of semantic mapping and post-processing map refinement. In semantic mapping, a point cloud is measured by the 3D Lidar and is transformed as globally consistent to generate a 3D map. In parallel, CNN-based 2D semantic segmentation is performed by using images obtained from a camera. Thereafter, incremental semantic labeling is performed to integrate the results from these two stages. After completing semantic mapping, map refinement is performed to improve the quality of the map. The purpose of this process involves erasing traces produced by moving vehicles and rectifying the spatial distribution of semantic information by correcting points where labels are projected erroneously. Figure 1 shows an example of the semantic map generated by our method.

### 2.1. Semantic Mapping

#### 2.1.1. Consistent Point Cloud Registration

In order to generate a global 3D map, it is necessary to transform the point cloud into corresponding odometry and subsequently register them with each other. Thus, it is necessary to accurately estimate each frame’s odometry including the position and angle in real-time. In the proposed approach, the odometry is obtained as a method to estimate the state by combining a GPS and an IMU. It is the most suitable method to reconstruct large-scale environments because it involves a low amount of computation and does not require structural features. In order to transform the point cloud, two elements are required, namely a rotation matrix and a translation vector. In this study, we use an expensive GPS and IMU that includes a filtering-based state estimation process. The sensor exhibits centimeter-level accuracy, and thus it is sufficient for 3D mapping [28]. Each point cloud is transformed as shown in (1), and the global 3D map is obtained by registering it.

(1) $${\hat{P}}_{i}=R_{i}\times P_{i}+T_{i}$$

where $R_{i}$ and $T_{i}$ denote a rotation matrix and a translation vector at the *i*th frame, respectively. $P_{i}$ is measured point cloud at the *i*th frame. Subsequently, the point cloud is changed into the voxel grid for memory efficiency and the incremental semantic labeling described in Section 2.1.3. The grid size is set to 5 cm × 5 cm × 5 cm. By performing the registration of these voxels, we generate a geometric 3D map.

#### 2.1.2. 2D Semantic Segmentation

**Figure 3:**
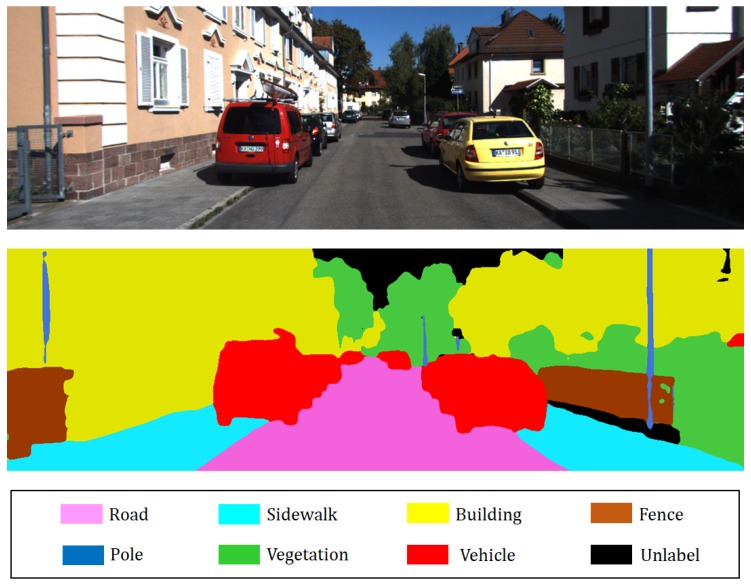
Example of 2D semantic segmentation: (Top) input image (Bottom) prediction.

Camera-based 2D semantic segmentation is performed to provide geometric 3D map semantic information. The reason why we exploit 2D semantic segmentation instead of 3D semantic segmentation is that 2D semantic segmentation is not only superior in performance but also requires short computation time to implement compared to 3D semantic segmentation. Recently, given that CNN-based segmentation approaches led to significant performance improvement, we selected RefineNet, which is a CNN model for 2D semantic segmentation [24]. RefineNet performs 2D semantic segmentation by using multi-resolution fusion and chained residual pooling, and results indicate that the RefineNet outperforms state-of-the-art methods. The reason for selecting the RefineNet as a 2D semantic segmentation tool is because it exhibits excellent performance and is available as an open source. We used a pre-trained model involving the Cityscape dataset and fine-tuned the model by using the Camvid dataset to modify it. In our framework, 2D semantic segmentation takes an image that has the size of W×H×3 as an input and returns the probability of labels per pixel that has the size of W×H×8. Here, *W* and *H* denote width and height of the image, respectively. Subsequently, 8 implies the number of semantic labels, including unlabeled category. The probability of labels per pixel is used for the incremental semantic labeling process, and Figure 3 shows the results of 2D semantic segmentation.

#### 2.1.3. Incremental Semantic Labeling

In order to construct a semantic map, it is necessary to integrate the location of the voxel with semantic information. In order to achieve this goal, we developed incremental semantic labeling that consists of the following three-stage pipelines: Coordinate alignment, Error minimization, and Semantic information fusion.

#### Coordinate Alignment

The first pipeline aligns the coordinate system between a 3D Lidar and a camera such that we can know the pixels that correspond to each voxel. This is achieved by converting the 3D Lidar coordinate system to the camera coordinate system by using an extrinsic parameter that describes the positional relationship between two sensors as shown in (2) and (3).

(2) $$\left[\begin{array}{c} u\\ v\\ w \end{array}\right]=P_{lidar}^{cam}\left[\begin{array}{c} x_{v}\\ y_{v}\\ z_{v}\\ 1 \end{array}\right]$$

(3) $$\left[\begin{array}{c} u_{c}\\ v_{c} \end{array}\right]=\left[\begin{array}{c} \frac{u}{w}\\ \frac{v}{w} \end{array}\right]$$

where $\left(x_{v},y_{v},z_{v}\right)$ denotes the coordinates of the voxel, and $\left(u_{c},v_{c}\right)$ denotes the pixel corresponding to $\left(x_{v},y_{v},z_{v}\right)$. Additionally, $P_{lidar}^{cam}=P_{cam}R_{rect}T_{lidar}^{cam}$ denotes the extrinsic parameter that converts the 3D Lidar coordinate system into the camera coordinate system. $P_{cam}$ is the projection matrix of a camera, and $R_{rect}$ denotes the rectifying matrix of the camera.

$$\begin{array}{c} T_{lidar}^{cam}=\left[\begin{array}{cc} R_{lidar}^{cam} & t_{lidar}^{cam}\\ 0_{1\times 3} & 1 \end{array}\right],\;R_{lidar}^{cam}\in R^{3\times 3},\;t_{lidar}^{cam}\in R^{1\times 3}. \end{array}$$

**Figure 4:**
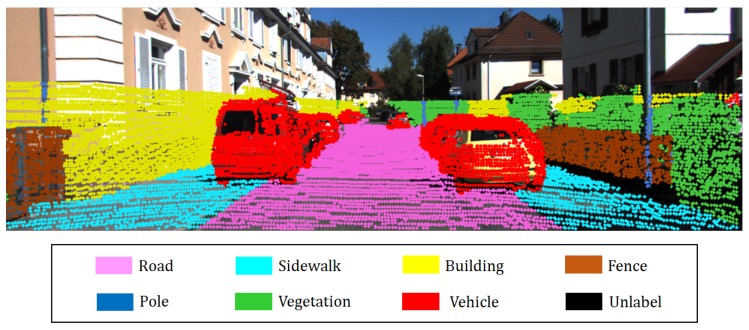
Coordinate alignment: labeled voxels are projected onto image.

$R_{lidar}^{cam}$ and $t_{lidar}^{cam}$ are the rotation matrix and translation vector from a 3D Lidar to the camera, respectively. Estimating the extrinsic parameter is a non-trivial task because the correspondences are difficult to establish. Nevertheless, several methods were introduced for extrinsic parameter estimation. For details, refer to [29,30]. By aligning the coordinate system, voxels with labels are created every frame for the error minimization process. Specifically, the label of each voxel is determined as the label with the highest probability, and the probability distribution for eight labels is stored in the voxel for semantic information fusion. Figure 4 shows the results of coordinate alignment.

#### Error Minimization

The labeled voxels generated by the coordinate alignment includes errors caused by projection error and inaccuracy of segmentation. In order to remove erroneously segmented voxels, a clustering method and a classifier are utilized. There have been previous studies that segment and classify a point cloud. In particular, several CNN-based approaches have been proposed, which take a point cloud and assign a semantic label to each point. Typical examples are PointNet [16], SEGCloud [17], and Semantic3D.net [31]. However, these methods require a long time to compute and are not suitable for our application. Furthermore, various segmentation algorithms and features-based classification algorithms have also been introduced, which have the advantage of low computational burden. Therefore, we adopted a method of combining a clustering method and a classifier. First, voxels with the same label that are spatially distributed are grouped by Euclidean clustering. Euclidean clustering is a method to create clusters for points within a certain distance, and it involves the advantage of requiring a low amount of computation. Generally, errors in the labeled voxels are spatially distant from a true object in 3D space, and thus Euclidean clustering is advantageous since it distinguishes false voxels from true objects. After the clustering process, we extract features that are suitable for the classification of the given clusters. There are no clear standard features for 3D data, and thus we use several different features. In this study, lengths of the X, Y, and Z axes as well as eigenvalue-based features [32] including linearity, planarity, scattering, omnivariance, anisotropy, and eigenentropy are utilized. Eigenvalue-based features are shown in (4).

(4) $$\begin{array}{cc} Linearity: & L_{\lambda }=\frac{e_{1}-e_{2}}{e_{1}}\\ Planarity: & P_{\lambda }=\frac{e_{2}-e_{3}}{e_{1}}\\ Scattering: & S_{\lambda }=\frac{e_{3}}{e_{1}}\\ Omnivariance: & O_{\lambda }=\sqrt[{3}]{e_{1}\;e_{2}\;e_{3}}\\ Anisotropy: & A_{\lambda }=\frac{e_{1}-e_{3}}{e_{1}}\\ Eigenentropy: & E_{\lambda }=-\sum_{i=1}^{3}e_{i}\ln\left(e_{i}\right) \end{array}$$

where $e_{1},e_{2}$ and $e_{3}$ denote the normalized eigenvalues of the cluster $C_{i}$. These features are used to erase clusters with incorrect semantic information in each frame. With respect to this operation, we opted for a learning-based classifier since it is difficult to classify it by using a simple expression.

**Figure 5:**
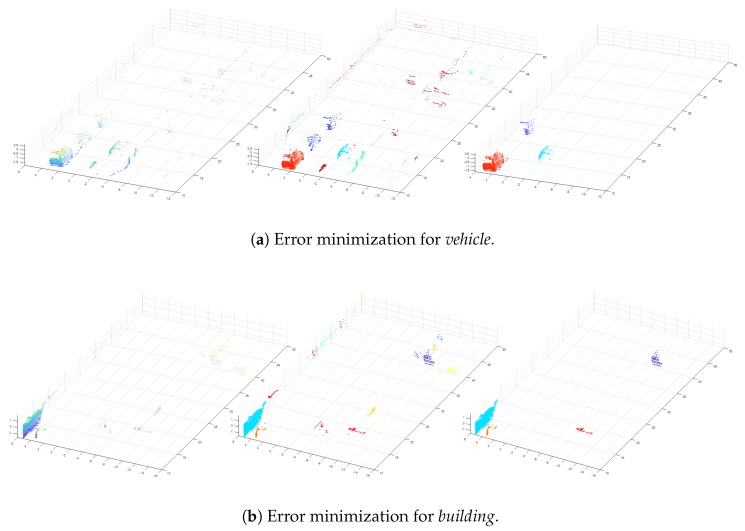
Result of the error minimization process. (First column) a set of voxels with the same label. (Second column) results of Euclidean clustering. (Third column) results obtained by the classifier.

We employ a random forest for its classification that consists of an ensemble of randomly trained decision trees and use it to vote for the winning class. The reason for selecting the random forest corresponds to its classification and timing performance. The random forest in our framework takes the feature vector of each cluster as an input and returns 0 (false) or 1 (true) as an output. We directly extracted positive and negative samples in 3D Lidar data for seven labels and trained each classifier by using them. Thus, seven trained classifiers are used in our system. This process removes wrongly segmented voxels in each frame, and Figure 5 shows an example of the result.

#### Semantic Information Fusion

Each voxel can be observed as a different label in each frame, and thus it is necessary to determine the final label for each voxel by fusing the observed labels. In order to achieve this, we update the probability distribution over the set of labels in the form of a recursive Bayesian update to incrementally reflect semantic information to the map. It is expressed as (5).

(5) $$p\left(l_{k}^{v}|I_{1:k},P_{1:k}\right)\approx p\left(l_{k}^{v}|I_{1:k-1},P_{1:k-1}\right)p\left(l_{k}^{v(u)}|I_{k},P_{k}\right)$$

where $l_{k}^{v}$ denotes the label of voxel, *v*, at *k*th frame, *I* denotes the image, and *P* denotes the point cloud. Furthermore, $p\left(\cdot \right)$ denotes the probability distribution and $l_{k}^{v(u)}$ denotes the semantic label of a voxel v(u) which corresponds to a pixel *u*. The first part of the right side is a prior probability distribution of voxel’s label that uses the probability distribution over the set of labels stored in each voxel. Additionally, the newly generated voxel is initialized with a uniform probability distribution. The latter part of the right side in (5) represents the probability distribution of the semantic label for each voxel given that $I_{k}$ and $P_{k}$ are obtained, which can be computed from 2D semantic segmentation, coordinate alignment and error minimization. Specifically, the probability distribution over the set of labels in each pixel is obtained from 2D semantic segmentation, and the coordinate alignment process determines which voxel corresponds to each pixel. Thus, we can transfer the probability distribution of the semantic label in each pixel to the corresponding voxel, and it is used as the latter part of the right side in (5). A posterior probability distribution in each voxel is maximized to assign the label with the maximum probability to the voxel as shown in (6). In (6), probabilities of eight labels are stored in each voxel, so the label with maximum probability is obtained by using a simple sorting method.

(6) $$l_{k}^{v}=\underset{l_{k}^{v}}{\mathrm{argmax}}p\left(l_{k}^{v}|I_{1:k},P_{1:k}\right)$$

where $l_{k}^{v}$ is one of eight labels, and the semantic map is extended incrementally with images and point clouds by using this operation.

### 2.2. Map Refinement

#### 2.2.1. Rectification of Label’s Spatial Distribution

A few voxels can be wrongly segmented even in a generated semantic map. However, these voxels are characterized as far from the true object and with low density. We adopted spatial reasoning to reduce these voxels. First, *K* nearest voxels of a voxel are detected, and the average distance between these voxels and a voxel is calculated. Subsequently, if the average distance is less than a certain threshold, then it corresponds to a well-segmented voxel, and otherwise it is considered as a noise voxel. It is expressed as follows:

(7) $$P^{v}=\left\{\begin{array}{cc} 1, & \mathrm{if}\;\mathrm{Avg}|P^{v}-P_{nn}^{v}|<\delta_{d}\\ 0, & \mathrm{otherwise} \end{array}\right.$$

where 1 and 0 denote well segmented and wrongly segmented, respectively. Additionally, $P_{nn}^{v}$ corresponds to *K* nearest voxels of $P^{v}$, and $\delta_{d}$ is determined experimentally. This process requires a low amount of computation, and thus the processing time is also low even if the size of the map increases. Additionally, it exhibits an advantage in terms of the performance. By using this process, we obtain a final semantic map with fewer erroneous voxels.

#### 2.2.2. Removal of Traces

**Figure 6:**
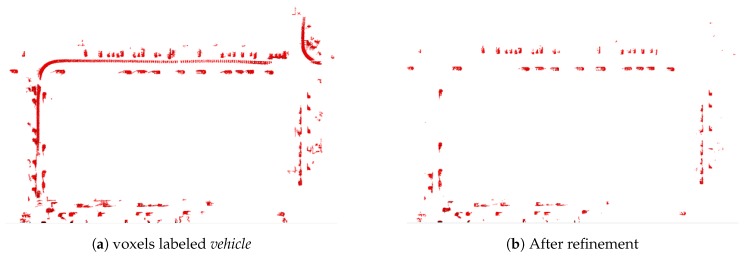
Example of the trace removal from the top view.

When the semantic map is completed, a number of traces are created by moving vehicles, and this makes the map messy. In order to improve the applicability of the semantic map, it is essential to remove these traces. In order to solve it, we extract the map corresponding to *vehicle* label, and apply density-based spatial clustering of applications with noise (DBSCAN) [33]. Specifically, DBSCAN is a clustering algorithm with low parametric characteristics that groups voxels based on spatial distribution. However, the disadvantage is that the memory efficiency is significantly reduced based on the size of the generated map, and thereby requires a long calculation time. In order to address the problem, we convert the 3D voxel representation to a 2D grid representation. This is achieved by projecting all 3D voxels to the same *Z*, and it is defined as follows:

(8) $$M_{2D}^{v}\left(x,y\right)=M_{3D}^{v}\left(x,y,z=0\right)$$

where $M_{2D}^{v}$ and $M_{3D}^{v}$ denote 2D grid and 3D voxel maps, respectively. Subsequently, DBSCAN is performed on 2D grids to create clusters based on the spatial distribution. Given the clusters, several constraints are applied to distinguish between moving and static vehicles. With respect to the clusters corresponding to the moving vehicles, many voxels are included and the length is relatively long when compared with the stationary vehicles shown in Figure 6a. Therefore, we classify moving vehicles and stationary vehicles using (9) by setting constraints based on the number of voxels and length.

(9) $$C_{i}=\left\{\begin{array}{cc} stationary, & \mathrm{if}\;\left(D_{i}<\eta_{d}\right)\wedge \left(L_{i}<\eta_{l}\right)\\ moving, & \mathrm{otherwise} \end{array}\right.$$

where $D_{i}$ denotes the number of voxels, and $L_{i}$ denotes the length of the *i*th cluster. Additionally, $\eta_{d}$ and $\eta_{l}$ denote the threshold values that are obtained experimentally. Finally, only stationary vehicles are left on the semantic map if we convert the 2D points corresponding to the stationary vehicles back to the 3D voxels. Figure 6 shows the results of this process.

## 3. Experimental Results

We compared our algorithm with state-of-the-art methods to objectively evaluate its performance. Ref. [13,15,27,34] were selected for comparison purposes because they performed 3D semantic mapping in an outdoor environment. The experiments were conducted by using the KITTI dataset that is publicly available [35]. Sequences in KITTI dataset were recorded with a 3D Lidar, cameras, GPS, and IMU in urban environments.

### 3.1. Dataset

We used three sequences to experiment, namely 15th sequence in the road category, 18th sequence in the residential category and 27th sequence in the residential category. The 15th sequence is a dataset of road environment with a duration of 30 s and a total of 303 frames. In the sequence, there are several moving vehicles and few structural features. The 18th sequence is 276 s long and records 2769 frames in a large-scale environment. Objects corresponding to the seven labels are evenly distributed in the sequence. Finally, the 27th sequence recorded a large-scale environment that describes the residential place. Its length is 111 s and it consists of 1112 frames.

### 3.2. Implementation Details

Our experiments were performed on a computer with Intel Core I7 (3.40 GHz) and NVIDA GeForce GTX 1080Ti, and we operated our algorithm by using Matlab. Furthermore, the sensor data used in the experiments is provided by the KITTI dataset. Specifically, a 3D Lidar that corresponds to HDL-64E manufactured by Velodyne, a camera model that corresponds to FL2-14S3C-C manufactured by PointGrey with a resolution of 1.4 Megapixels, and a model of GPS/IMU that corresponds to OXTS RT 3003 with a resolution of 0.02 m and 0.1 degree are used. In our experiments, we used the points that exceeded 5 m and were less than 50 m in distance. This is because points are less reliable if the distance exceeds or is less than a certain threshold, and 2D semantic segmentation is poor at the pixels that are too close or far apart from the camera. Finally, in the map refinement, the number of nearest voxels, *K*, was set to 6, and $\delta_{d}$ was set to 20 cm. Additionally, $\eta_{d}$ and $\eta_{l}$ were set to 500 and 10 m, respectively. The parameters were empirically obtained by performing several experiments.

### 3.3. Qualitative Evaluation

**Figure 7:**
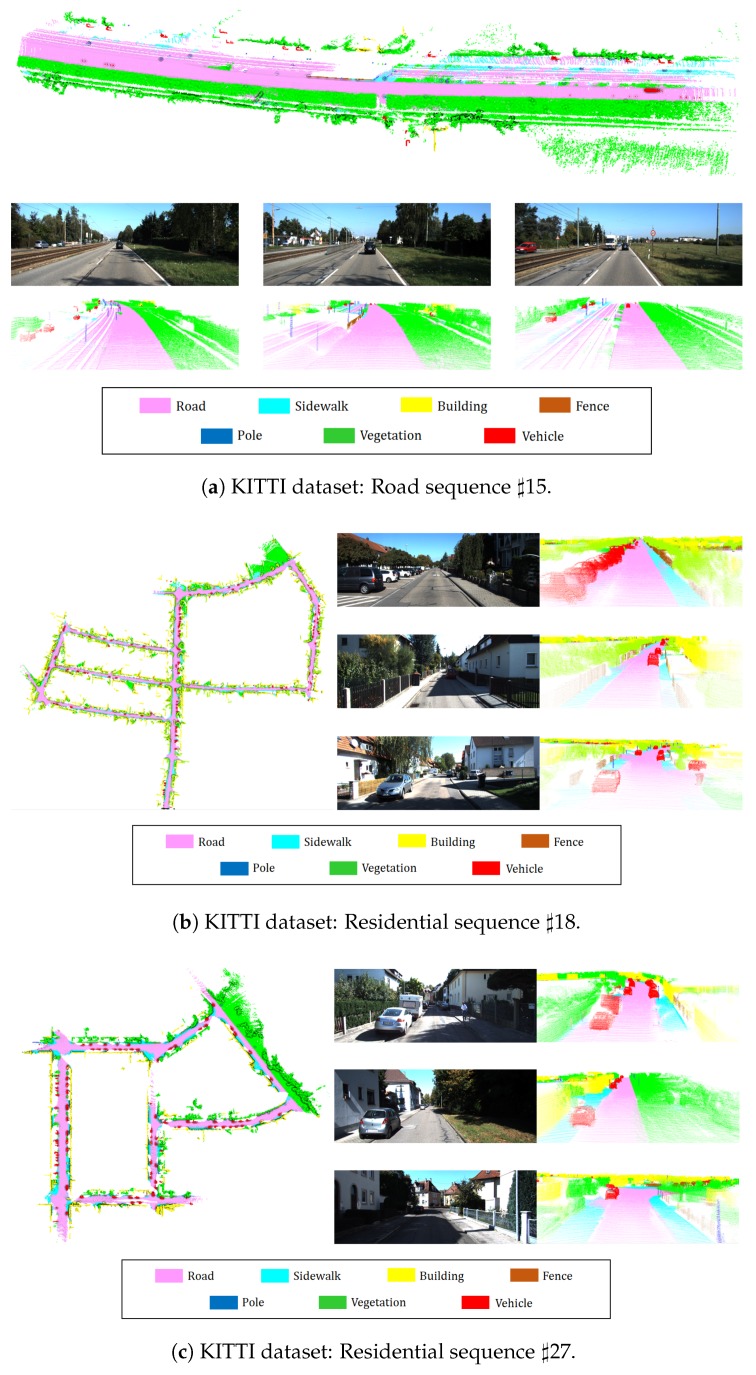
Visualization of semantic 3D mapping results. Top view for the entire map and three close-up views with different scenarios.

**Figure 8:**
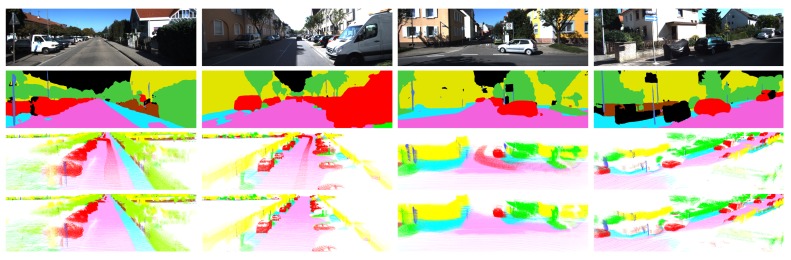
Effectiveness of the map refinement. (First Row) original images. (Second Row) 2D semantic segmentation. (Third Row) semantic 3D map without map refinement. (Bottom Row) semantic 3D map with map refinement.

**Figure 9:**
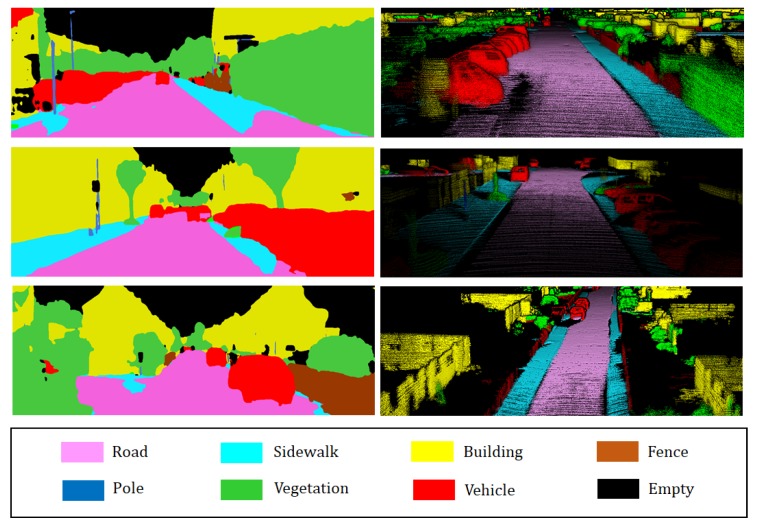
Comparison of 2D semantic segmentation and 3D semantic segmentation.

We first present some qualitative results of semantic 3D mapping in Figure 7. It shows a top view of the entire semantic map and three close-up views of different scenarios for each sequence. The qualitative results indicate that our system correctly assigned the label in challenging conditions and successfully performed 3D reconstruction in large-scale environments. The proposed algorithm performed 3D segmentation well even with respect to thin poles. However, there is a disadvantage in the proposed method as shown in Figure 7a. In our system, a railroad is not trained in the 2D semantic segmentation, and thus our system classified it as the most similar thing among seven labels. Therefore, a railroad in the middle of the map was classified as *road* and *sidewalk* in our semantic map. This problem will be overcome in the future by including additional labels in the CNN training stage. Conversely, with respect to case objects corresponding to the trained seven labels that occupy most of the environment, such as those shown in Figure 7b,c, it is possible to generate an accurate semantic 3D map. Additionally, although most projection errors caused by overlapped objects are unavoidable, the results indicate that projection errors are reduced due to the use of incremental semantic labeling and the map refinement process. Consequently, we demonstrated the superiority of our method by successfully performing semantic mapping in various environments. Figure 8 shows the effectiveness of the map refinement. If the map refinement is not applied, then traces created by moving vehicles remain on the map. However, by using the map refinement, we remove traces while preserving the stationary vehicles. Furthermore, the spatial distribution of labels is modified by effectively erasing voxels that were not removed by using error minimization. Map refinement improves 3D segmentation performance and is beneficial for other applications. In addition, we would like to highlight that 3D segmentation shows better results than 2D segmentation. As shown in Figure 9, some pixels were segmented wrongly in 2D segmentation results but they were segmented correctly in 3D segmentation results.

### 3.4. Quantitative Evaluation

In this section, we demonstrate the superiority of our method by quantitatively evaluating 3D semantic segmentation accuracy with those of state-of-the-art methods. As mentioned in Section 3, we used [13,15,27,34] to evaluate the performance of our algorithm because it was proven that they yielded good results in terms of generating a semantic 3D map in outdoor environments. Ref. [13,15,27,34] are camera-based approaches. We adopted a standard metric of label accuracy and intersection over union (IoU) to evaluate the performance. The indexes are defined as follows:

(10) $$\mathrm{Accuracy}=\frac{\mathrm{TP}}{\mathrm{TP}+\mathrm{FP}}$$

(11) $$\mathrm{IoU}=\frac{\mathrm{TP}}{\mathrm{TP}+\mathrm{FP}+\mathrm{FN}}$$

where TP, FP, and FN stand for True Positive, False Positive, and False Negative, respectively. Table 1 presents the comparison results in terms of accuracy and IoU. The results of other studies are directly obtained from the paper, and ‘−’ indicates that the number is not given. For a detailed experimental environment, refer to the relevant paper. For the purpose of fairness, our algorithm is also evaluated on the Sengupta labelled dataset [34] that is an experimental environment for other algorithms. As shown in Table 1, our method outperformed other state-of-the-art methods for most labels. With respect to *vegetation*, our method exhibited slightly poorer performance when compared with that of [15], although it exhibited a better performance relative to the remaining labels. With respect to the label *sidewalk*, there was an increase in the accuracy by approximately 1.4%. With respect to *building*, *fence*, *pole* and *vehicle*, we achieved 1.1%, 7.4%, 10.9% and 2.8% improvement, respectively. The proposed method showed better results in IoU as similar to accuracy. In terms of IoU, our algorithm exhibited the best performance for the six labels. There was a performance improvement of 0.1% for *road*, 1.3% for *sidewalk*, 3.0% for *building*, 1.9% for *fence*, 2.4% for *vegetation*, and 1.4% for *vehicle*. However, in case of *pole*, IoU was reduced by 17.8% in terms of its accuracy. The reason for this is that there are a few points for *pole* in the dataset, so some erroneous points have a big influence on the performance evaluation. With respect to the remaining labels, IoU is similar in terms of the accuracy, and this demonstrates superiority of our algorithm. There are three main reasons as to why our method outperforms existing methods. First, we opted for the RefineNet as a 2D semantic segmentation tool. It exhibited a better performance when compared with those of other semantic segmentation methods. Second, error minimization processes including clustering, feature extraction, and classifiers help in improving the performance. There are well-trained classifiers for each label, and thus wrongly segmented voxels are erased. Finally, we developed map refinement that modified the spatial distribution of labels and effectively removed traces.

### 3.5. Time Analysis

We also provide a time analysis of components in our framework. As shown in Table 2, there are four main components in our system, namely 2D semantic segmentation, Euclidean clustering, random forest, and semantic information fusion. Map refinement is processed in a batch for the generated semantic map that is dependent on the size of the generated map, and thus, the map refinement is not included in Table 2. The first three parts utilize open source and public libraries. Most of the time entailed in our algorithm is spent on the first three parts, and they were implemented in Matlab. The last part corresponds to semantic information fusion, and it requires less computation time because it contains only simple multiplications. As shown in Table 2, our algorithm runs at 2 Hz in Matlab and can be faster by using a low channel Lidar or a camera with low resolution, and implementing the algorithm in other coding environments. The map refinement consists of two major parts, and we provide time complexity of them. With respect to the rectification of label’s spatial distribution, most of the processing time is spent in determining *K* nearest neighbor voxels, and its time complexity is a form of O(KN+ND) that depends on the number of nearest voxels, *K*, dimensionality, *D*, and the total number of voxels, *N*. There are several possible ways to improve the speed. In order to achieve it, we can reduce the number of nearest voxels, *K* or increase grid size to reduce the total number of voxels. However, these could increase the segmentation error and may not qualify for other purposes. With respect to the removal of traces, DBSCAN occupies the highest part of the processing time, and its complexity is expressed as $O(N_{2}logN_{2})$ where $N_{2}$ denotes the number of points in 2D grids. In order to reduce the computation time in this part, we can increase the grid size to reduce the number of points. However, it leads to the generation of a sparse 3D map and increases the error. However, the map refinement is processed in a batch after semantic mapping, and thus, it is more important to improve the quality of the map than to shorten the computation time.

## 4. Conclusions

In this study, we proposed a method to generate a semantic 3D map in an urban environment by combining a 3D Lidar and a camera and involving the use of semantic mapping and map refinement. With respect to semantic mapping, a GPS and an IMU are fused for a localization, and point clouds obtained from the 3D Lidar are registered into a 3D map. Subsequently, 2D semantic segmentation is performed, and it is integrated with point clouds. Additionally, Euclidean clustering, feature extraction, and classifiers are consecutively used to minimize the 3D segmentation error. Furthermore, a recursive Bayesian update scheme is utilized to handle multiple observations. The map refinement takes the generated semantic map as an input and enhances the quality of the map. To rectify the spatial distribution of labels, wrongly segmented voxels are erased by comparing them with nearest neighbor voxels. Furthermore, the DBSCAN method is utilized to remove traces, and we used a strategy that converts a 3D representation into a 2D representation to reduce the computational burden. The proposed method is compared with the latest algorithms for challenging sequences to demonstrate the superiority of the method.

It is expected that the results of our study can be applied to various applications such as robot navigation and surveying. A future study will expand the results to semantic mapping in an indoor environment and will explore a semantic SLAM in which SLAM and semantics benefit each other.

## Figures and Tables

**Table 1:** Quantitative results for 3D semantic segmentation on the Sengupta labelled dataset. The bold fonts indicate the best results.

	Method	Road	Sidewalk	Building	Fence	Pole	Vegetation	Vehicle
Accuracy	Sengupta [34]	97.8	86.5	88.5	46.1	38.2	86.9	88.5
	Sengupta [27]	97.0	73.4	89.1	45.7	3.3	81.2	72.5
	Vineet [13]	**98.7**	91.8	97.2	47.8	51.4	94.1	94.1
	Yang [15]	**98.7**	93.8	98.2	84.7	66.3	**98.7**	95.5
	Ours	**98.7**	**95.2**	**99.3**	**92.1**	**77.2**	95.3	**98.3**
IoU	Sengupta [34]	96.3	68.4	83.8	45.2	28.9	74.3	63.5
	Sengupta [27]	87.8	49.1	73.8	43.7	1.9	65.2	55.8
	Vineet [13]	94.7	73.8	88.3	46.3	41.7	83.2	79.5
	Yang [15]	96.6	90.0	95.4	81.1	**61.5**	91.0	94.6
	Ours	**96.7**	**91.3**	**98.4**	**83.0**	59.4	**93.4**	**96.0**

**Table 2:** Time analysis of the proposed algorithm.

Method	Time(s)
2D Semantic Segmentation	0.2412
Euclidean Clustering	0.0898
Random Forest	0.1913
Semantic information fusion	0.0003
